# Menage a Quoi? Optimal Number of Peer Reviewers

**DOI:** 10.1371/journal.pone.0120838

**Published:** 2015-04-01

**Authors:** Richard R. Snell

**Affiliations:** Research Knowledge Translation and Ethics Portfolio, Canadian Institutes of Health Research, Ottawa, Ontario, Canada; University of Cape Town, SOUTH AFRICA

## Abstract

Peer review represents the primary mechanism used by funding agencies to allocate financial support and by journals to select manuscripts for publication, yet recent Cochrane reviews determined literature on peer review best practice is sparse. Key to improving the process are reduction of inherent vulnerability to high degree of randomness and, from an economic perspective, limiting both the substantial indirect costs related to reviewer time invested and direct administrative costs to funding agencies, publishers and research institutions. Use of additional reviewers per application may increase reliability and decision consistency, but adds to overall cost and burden. The optimal number of reviewers per application, while not known, is thought to vary with accuracy of judges or evaluation methods. Here I use bootstrapping of replicated peer review data from a Post-doctoral Fellowships competition to show that five reviewers per application represents a practical optimum which avoids large random effects evident when fewer reviewers are used, a point where additional reviewers at increasing cost provides only diminishing incremental gains in chance-corrected consistency of decision outcomes. Random effects were most evident in the relative mid-range of competitiveness. Results support aggressive high- and low-end stratification or triaging of applications for subsequent stages of review, with the proportion and set of mid-range submissions to be retained for further consideration being dependent on overall success rate.

## Introduction

Peer review of proposals submitted to agencies for funding support and manuscripts submitted to journals for publication has been termed “a cornerstone of science” [1], “the cornerstone of quality assurance” [2], “the most commonly used method for evaluating scientific research” [3] and “the gold standard for evaluating scientific merit” [4]. In contrast, recent Cochrane reviews determined literature on evidence-based best practice in peer review is sparse [5,6]. Typically, most research funding agency peer review involves a contextual framework [7] of committee-based evaluation processes and, increasingly, fewer or no face-to-face meetings [8] to assess proposals and inform ‘yes-no’ decisions for research support [9]. Reported limitations to peer review include low inter-rater reliability [3,10,11], low reproducibility [12–14], low predictive validity [15–17], potentially limited impact from discussion on scores or outcomes [4,8], potential bias [18–20], potential conservatism [21,22] and risk aversion [16,18,22,23]. However, proposed alternatives to peer review such as sandpit methods or workshop review [24], community-based evaluation [25], or collective reallocation [26], have only been rarely attempted, reflecting (at least in part) a general lack of evidence of the effectiveness or efficiency of alternatives and also the difficulty of shifting the prevailing decision paradigm for peer review [15].

The overall number of proposals submitted for potential support is increasing for most research funding agencies [9,27], and funders are increasingly overburdened by workload and complexity of the review process. With more applications, there is concomitant growing pressure for the recruitment of more peer reviewers, wherein reviewer participation appears largely driven by motives related to reciprocity, social norms within the scientific community [28], scientific quality control, communal obligation and self—interest [29]. However, despite these strong motives, peer reviewers are becoming a scarce resource: the work is generally unpaid or recompensed with only modest honoraria, and many potential recruits also cite the dual burden of conflict with other work plus lack of time [30] as legitimate reasons to not participate in peer review. Total cost and burden of peer review is directly proportional to the number of reviewers assigned per application. Thus, determining the minimum number of reviewers per application that will inform reliable and consistent decisions, especially given the practical difficulty of increasing the reviewer count per application, is a key operational consideration for research funders, reviewers and applicants alike.

Little is known about variation in decision outcome with incremental increase in numbers of reviewers across a range of applications submitted to actual competition. Bootstrapped estimates of outcomes, using retrospective data from an Australian grants competition [23], provided evidence that the proportion of proposals “sometimes” funded was lowest with larger simulated committees, leading to the conclusion that “larger panels are better than smaller ones.” Estimates of up to 38,416 reviews per grant application to distinguish peer review scores at an acceptable level of precision [31], suggested unrealistic numbers of reviewers would be required in an ideal peer review system.

Here I use bootstrapping of replicate reviewer scores, from a Canadian Institutes of Health Research (CIHR) spring 2013 post-doctoral Fellowships competition for biomedical applicants. Consistency was estimated in decision outcomes with incremental increases in number of reviewers per applications. Guidelines are proposed for the minimum number of reviewers required per application and for triaging depending on competition success rate.

## Methods

### Data source, rating methods and peer review

Prior to assignment of reviewers to applications, a subset of 100 applications (of 406 in the competition) was randomly selected for replicate review. CIHR’s standard peer review methodology for Fellowships competition, as of early 2013, included assignment of each application to 3 reviewers (referred to herein as a reviewer triad), randomly selected from all available committee members subject to the following constraints: exclusion of conflicts of interest, balancing reviewer workload and limiting the number of assignments per reviewer, and language matching (i.e., applications to CIHR may be in French or English and unilingual reviewers received applications only in their own language). For this competition, an additional constraint was used: exclusion of reviewers from more than one triad for any single application (no reviewer reviewed any application more than once).

Each of the 100 applications was reviewed by three randomly assigned reviewer triads (subject to the constraints outlined above), producing nine independent “at—home” pre—scores per application (which comprised the source data for this study). Reviewers scored multiple criteria on applications, on a scale of 0 [least competitive] to 4.9 [most competitive] (i.e., 50 possible states). Reviewers provided written evaluations and pre—scores through “ResearchNet” (CIHR’s Internet—enabled peer reviewer interface). Following completion of peer review, pre—score data (averaged by reviewer, for each application) were extracted from CIHR’s electronic database (anonymized data provided, S1 Data).

Although referred to as a “committee,” the pool of reviewers in this Fellowships competition participated entirely through ResearchNet with no face-to-face meeting of the committee as a whole. This review process entailed a series of discrete reviews by non—associated reviewer triads, some with overlapping memberships, that otherwise did not interact. After each triad member submitted their pre-scores and reviews through ResearchNet, the input of their other triad members became accessible. Reviewers were offered the option of eDiscussion through an electronic asynchronous discussion tool, for the exchange of comments, sharing of reviews and display of each other’s scores. Not all triads engaged in eDiscussion. Not all reviewers read the reviews of other reviewers. Final scores were submitted following the eDiscussion stage, adjusted as each reviewer deemed appropriate on the full range of the scale.

This study was based on independent pre-scores rather than final scores informed either by eDiscussion within reviewer triads or the opportunity to read the reviews of other reviewers in the triad. Previous study of CIHR Fellowships competition [8] provided evidence that “[…] committee discussion and rating of proposals offered no improvement to fairness and effectiveness over and above that attainable from the pre-meeting evaluations." Similar to previous results [8], ca. 70% of final scores provided by reviewers were unchanged from their initial pre-scores. Outcomes based on pre-scores are strong predictors of final outcomes.

### Data Manipulation

For each bootstrap iteration, pre-score data were resampled with replacement, for each application, to simulate 20 step-wise increments of N_reviewers_ (i.e., N from 1 to 21). At each iteration, for each application and N_reviewer_ combination, each set of resampled scores (from 1 to N) was averaged and converted to a percentile rank within the 100 applications. Applications with tied average scores, as determined in each iteration from resampled data, shared their maximum percentile rank. Within each increment of N_reviewers_, percentile rank of each application was used to determine the modeled binary competition outcome (i.e., success or not) by comparison with thresholds that represented each of five success rate scenarios (i.e., the top 5%, 15%, 25%, 35% and 50% of applications were assigned ‘1’ rather than zero). These five scenarios spanned the range of success rates commonly encountered at research funding agencies (historically, for higher success rates). The pre-score resampling process was repeated over 10,000 iterations, generating a cumulative distribution of success probability for each application across increments of N_reviewers_, for each success rate scenario. Success probability distributions of the 100 applications, for each success scenario, were collectively represented as 3D histograms.

To assess the impact on overall chance-corrected decision consistency, as a result of incremental increases to N_reviewers_, Cohen’s kappa [32] and significance levels were calculated across binary competition outcomes of the 100 applications, for each pairing of N and N+1 resampled reviewers, within each success rate scenario. To reduce sampling bias in bootstrapped estimates of kappa and significance, and to enable calculation of Monte Carlo error level [33], kappa statistics were recalculated (10,000 iterations). R version 3.1.0 and 3.1.1 [34] was used for calculations, data resampling, bootstrapping and graphs (R code for analyses and graphics provided, S1 File).

### Ethics Statement

Research and analytical studies at CIHR fall under the Canadian Tri—council Policy Statement 2: Ethical Conduct for Research Involving Humans [35]. This study had the specific objective of quality assurance and quality improvement related to design elements and design assumptions within CIHR’s ongoing reform of its Open programs [36], and thus fell under Clause 2.5 of TCPS-2 and not within the scope of Research Ethics Board review in Canada. Nevertheless, reviewers were informed through ResearchNet, in advance of peer review, that CIHR would be evaluating its own processes, review would be replicated for a randomized sample of applications, and the majority of ratings and reviews would be used for decision purposes. All reviewers provided their electronic consent; no potential reviewer refused to provide consent.

For each of the 100 application with replicate reviews, one triad was randomly selected (within ResearchNet), prior to peer review, as the ‘active’ triad that would be used to determine the actual competition outcome (i.e., the decision to fund or not). Thus, prior to peer review, a specific set of 100 triads, among all possible triad combinations (i.e., 1 of 3^100^ possibilities), along with 306 additional triads (representing review of non-replicated applications) was identified for decision purposes. Throughout the competition process, neither CIHR staff nor reviewers were aware of which triads were ‘active’. Reviewers were unaware of which applications had replicate review. Applicants were unaware of this background study on number of reviewers which did not affect their application outcome.

## Results

**Fig 1:**
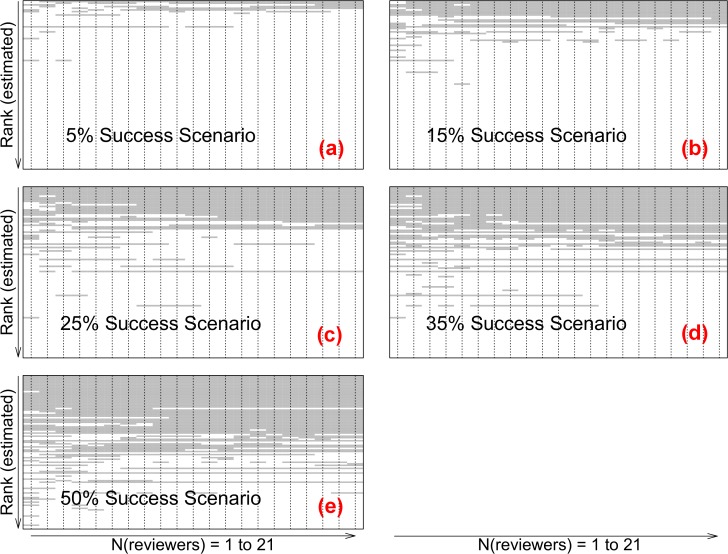
Simulated competition outcomes from a single bootstrap iteration (1 to 21 reviewers per application). Horizontal sequences represent simulated outcomes for each of 100 Fellowships applications (‘grey’ representing success) with incremental addition of reviewers, within different overall success rate scenarios. For each N to N+1_reviewers_, an additional score was sampled (with replacement) from 9 independent assessments. Discontinuities in horizontal grey/white—coding reflect changes in decision outcome with addition of a single reviewer. Within each iteration (representing one simulated competition), the outcome for many applications was invariant where N > 2, regardless of the success rate scenario.

Simulated competition outcomes for each application (Fig. 1), within a single bootstrapped iteration, are provided for N_reviewers_ (1 to 21) in each success rate scenario [(a) 5%, (b) 15%, (c) 25%, (d) 35% and (e) 50%]. Applications were sequenced by estimated rank (a gradient of relative competitiveness), based on the bootstrapped mean (10,000 iterations) of each set of 9 independent pre—scores per application. Within single iterations, outcomes for many applications were invariant, regardless of N_reviewers_ [i.e., continuous horizontal bands, of grey or white, represented continuous success (or not) respectively for individual applications with increased N_reviewers_]. For other applications, one or more additional reviewers per application resulted in one or more decision changes. Applications with higher variability among reviewer scores and especially those closer to the ‘payline’ of a particular scenario (i.e., applications with a rank closer to the cutoff of a specific success rate scenario) were more likely to exhibit decision reversals shown by horizontal pattern discontinuities.

**Fig 2:**
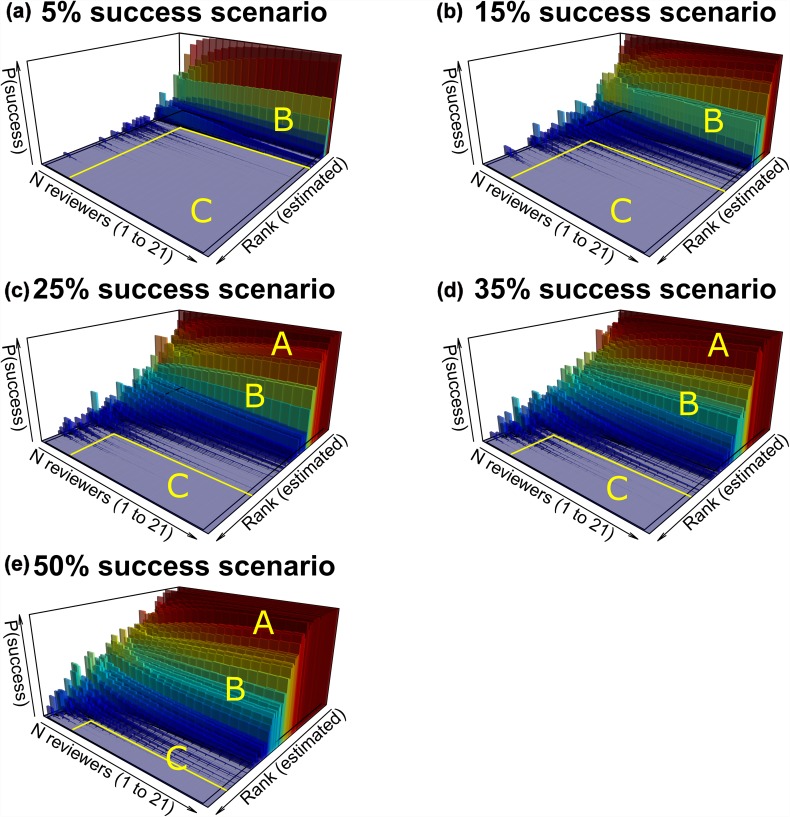
Funding success probability profiles in five overall competition scenarios (1 to 21 reviewers per application). Cumulative sum of 10,000 bootstrapped simulations of competition outcomes, for 100 applications within five overall success scenarios [5% (a), 15% (b), 25% (c), 35% (d), and 50% (e)]. Within some scenarios, the most competitive applications had ≥ ~95% probability of success (Category A). Applications of intermediate competitiveness (Category B) had a probability of success which varied from ~5% to ~95%. The least competitive applications (Category C) were rarely (≤ ~5% probability) or never successful.

The 3D distribution of probability of success for the 100 applications, across estimated rank (Fig. 2), with varying numbers of reviewers per application, varied among the five overall success rate scenarios. Applications were categorized into three outcome groups, based on cumulative probability of success over 10,000 simulated competition outcomes. Within each overall success scenario, ‘Category A’ represented highly competitive applications with elevated probability of success (i.e., ≥ ~95%). The relative proportion of Category A applications increased with overall competition success rate. ‘Category B’ represented less competitive applications, where the probability of success ranged from > ~5% to < ~95%. The relative proportion of Category B applications increased with overall competition success rate. Conversely, ‘Category C’ represented applications with low probability of success (i.e., ≤ ~5%). The relative proportion of Category C decreased with increased overall competition success rate. Category A applications were not identified in the 5% and 15% overall competition success rate scenarios (most applications were Category C).

**Fig 3:**
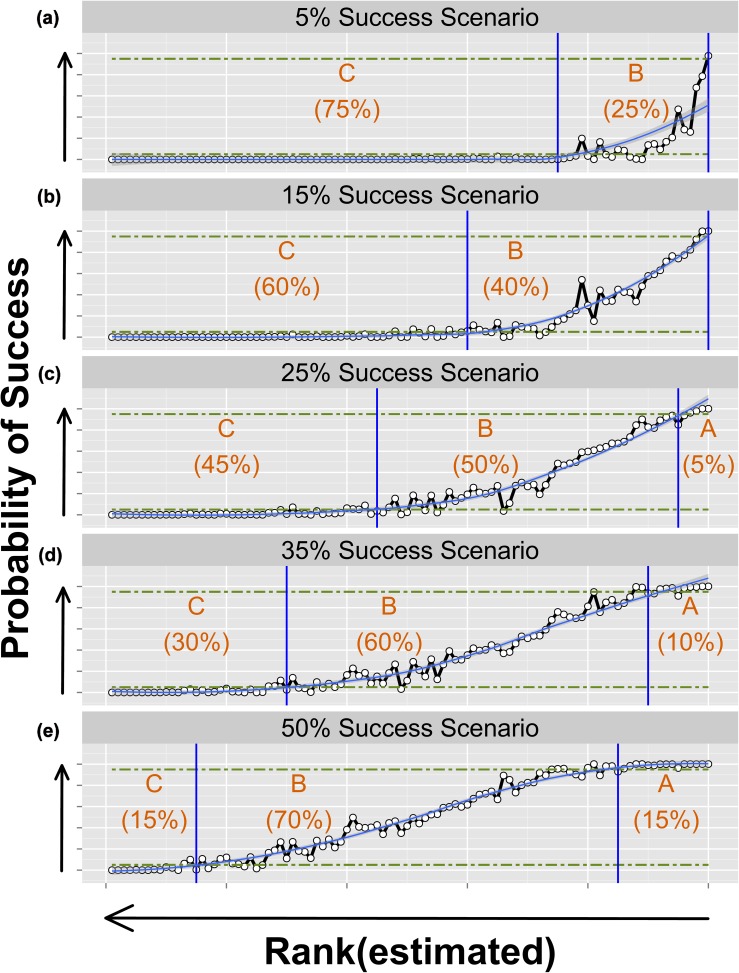
Relative proportion of applications in three outcome categories, in five overall competition scenarios (5 reviewers per application). For 100 applications within five overall competition success scenarios [5% (a), 15% (b), 25% (c), 35% (d), and 50% (e)], different proportions of applications had very high (A), medium (B) or very low (C) probability of success (categories defined in text) over 10,000 iterations of simulated competition results. Horizontal green lines indicated a probability of success of 5% and 95%. Vertical blue lines indicated where locally weighted polynomial regression (LOESS smoothing) curves intersected the 5% or 95% probability of success, or reached the highest ranked application (the limit of the graph).

Proportions of applications in Categories A, B and C, in five overall success scenarios, were further compared for N_reviewers_ = 5. Using locally weighted polynomial regression (LOESS smoothing in R [34]), probability of success for the 100 applications was calculated in relation to estimated rank. Within each success rate scenario, the proportion of applications (to the nearest 5%, based on the smoothed regression) which fell in each category was identified (Fig. 3).

**Fig 4:**
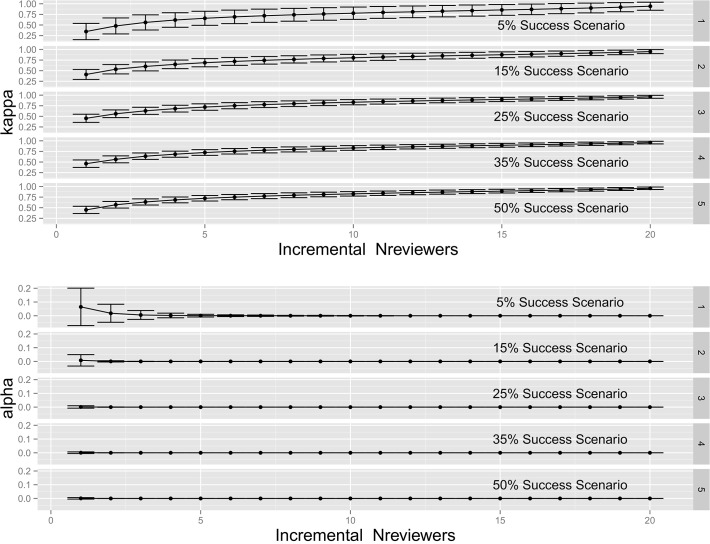
Bootstrapped kappa statistics of peer review decision consistency with incremental N to N+1 reviewers. In simulation of CIHR Fellowships competition outcomes, overall decision consistency improved with incremental addition of reviewers regardless of overall success rate scenario. Monte Carlo error analysis (standard deviation of bootstrapped estimates of kappa coefficients) [33] indicated broad overlap among incremental kappa values with increased reviewers. Kappa levels > 0.8 represented “almost perfect” consistency [32]. Kappa values were significant (α < 0.05) except in the 5% success scenario at the 1–2 and 2–3 reviewer increment.

Bootstrapped estimates of kappa and significance (alpha) levels, including Monte Carlo error estimates (i.e., as error bars) are shown (Fig. 4). Using benchmarks for kappa [32], there was “moderate” (0.41–0.60) to “substantial” (0.61–0.80) decision consistency, even at the 1–2 reviewer increment, although negative error bars extended to only “slight” agreement with the fewest reviewers. The 4–5 and 5–6 reviewer increment negative error bars extended to “moderate” agreement, rising to “substantial” at the 10–11 reviewer increment. Within increasing kappa, negative error extended to “almost perfect” (0.81–1.0) decision consistency at the 20–21 reviewer increment. Bootstrapped estimated of kappa were significant (α < 0.05) except for the 1–2 reviewer increment in the 5% success scenario. Positive Monte Carlo error bars for alpha levels extended above α = 0.05 (i.e., NS), for the 1–2 and 2–3 reviewer increment in the 5% success scenario but otherwise were within the range of significance (α < 0.05). Across all success rate scenarios, additional reviewers per application provided increased (though increasingly modest) improvement in overall decision consistency.

**Fig 5:**
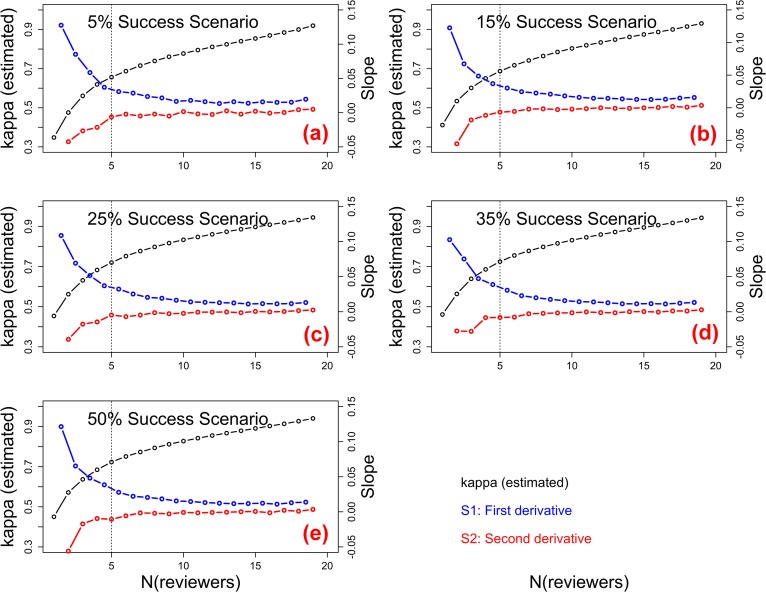
First derivative (S1) and second derivative (S2) of kappa, within incremental N to N+1 reviewers, across five overall competition success rate scenarios. Relative improvement of kappa reached stability at 4–5 reviewers per application or shortly thereafter, across all overall success rate scenarios [5% (a), 15% (**b**), 25% (c), 35% (d), and 50% (e)]. Vertical dashed lines represent the approximate S2 asymptotes.

To identify a meaningful (and practical) upper limit to N_reviewers_, several criterion—setting stopping—approaches [37] were tried (e.g., Scree plot methods used to identify a meaningful number of Principal Components). The most pragmatic (or otherwise useful) stopping—approach was based on a method to select sampling duration using second derivatives [38]. The first derivative local slope (S1) calculated from point—to—point local changes in kappa and second derivative local change in slope (S2) similarly calculated from point—to—point changes in S1, are shown (Fig. 5). Buffin—Bélanger & Roy [38] used natural logarithm transformation of the x—axis (time, covering many orders of magnitude), contributing to their distinct S2 inflection point. S2 of non-transformed kappa data in this study attained an asymptote at, or slightly, above the 4–5 reviewer increment in all success rate scenarios. Substantial levels of decision consistency (kappa ≥ 0.61) were achieved with 4–5 reviewers per application, for all success rate scenarios.

## Discussion and Conclusion

Variability among scores for an individual application, or among scores for applications close to it in terms of rank, yielded alternate score combinations (in different iterations) that frequently changed relative ranking. Depending on the success rate scenario and the proximity of an application to the threshold cutoff, changes in an application’s rank were often sufficient to reverse the simulated decision outcome. Even within single iterations (Fig. 1), an ‘invariant’ competition outcome for all applications was not achieved under any of the five funding scenarios as a result of adding additional reviewers per application, and ‘invariant competition outcomes’ do not appear to represent a realistic target or expectation for research funding agencies, reviewers or applicants. As has been identified in previous competition environments [4,10–14,23], some degree of alternative decision outcomes would be anticipated with re—running or replication of many or most competitions.

Classification of most applications to category A, B or C depended on success rate scenario. Variable outcomes were notable in Category B (Figs. 2, 3). Particularly where the overall competition success rate was low (i.e., 5% or 15%), the peer review process appeared highly effective in identifying applications with low probability of success (Category C). The process appeared far less effective in identifying noncompetitive Category C applications where the success rate was high (i.e., 35% or 50%). The previous observation, “[…] that reviewers have much less difficulty in agreeing on rejection than on acceptance” [10], may thus be linked to overall competition success rate.

Variable outcomes for many applications, regardless of category, were exacerbated where numbers of reviewers per application was low (especially N_reviewers_ ≤ 3, Fig. 2). With few reviewers, there was increased probability of negative decisions even for the most highly ranked Category A applications, and an increased probability of positive decisions for many of the least competitive applications (Category C). In other words, especially with few reviewers, “luck” [12] played an increased role in determining outcome for many applicants, particularly in the mid—range of competitiveness. Given larger error bars for kappa statistics, with low numbers of reviewers per application, and given that success rates are dropping for funding agencies [27], these results are supportive of the need to increase reviewer numbers to beyond 2 or 3 reviews per application.

Implications for stratifying applications (i.e., triaging) are considered in the context of the diversity of profiles for probability of success over different success rate scenarios with 5 reviewers per application (Fig. 3). In a multi-stage peer review process, such as would typically involve “at-home” review [8], input from reviewers can be acquired that is sufficient to place applications into rank order sequence for the purposes of stratification. Varying proportions of applications could be stratified into A, B and C categories, as in Table 1, depending on overall competition success rate. High and low rank applications (Fig. 3) could be excluded from further review with a decision to fund (Category A) and to reject (Category C), respectively, at this mid-point in the process. The Category B subset could be assigned to a subsequent phase of consideration (such as asynchronous online discussion, or potentially face-to-face discussion in some instances).

**Table 1:** Guidelines for triaging.

	Stratification of Applications (% by Category) within Gradient of Competitiveness^a^		
**Overall Competition Success Rate Scenario (%)**	C (Reject without further review)	B (Further review required)	A (Success without further review)
**5**	75	25	0
**15**	60	40	0
**25**	45	50	5
**35**	30	60	10
**50**	15	70	15

^a^Gradient of Competitiveness established by ranking applications on the basis of average (or bootstrapped average) raw scores or score percentiles (as appropriate for any given competition).

Stratification (percent by category) could be determined on a competition—by—competition basis, at the time applications are received, or, when application volume and competition success rate is otherwise determined (such as through a quota system with a predetermined number of eligible applications), thereby limiting expenditure of additional resources and reducing reviewer burden. Other peer review contexts of *ex ante* review in advance of performance (e.g., of research project proposals) and *ex post* retrospective review (e.g., of manuscripts for publication) [39] may similarly benefit from higher levels of decision consistency with increase to N_reviewers_ = 5.

Unlike competitions for fellowships, normally restricted to one application per applicant, many grant competitions function without intake limits. In such competitions, applicants are already incentivized to submit as many proposals as possible as a rational strategy to individually maximize the probability of funding, in an equivalent of game theory’s Prisoner’s Dilemma [27]. An optimal strategy, which could minimize both individual applicant and the collective total workload would be for applicants to cooperate by submitting one or a small number of applications per competition [27]. However, the element of chance in the decision process, evident in the 3D success probability profiles (Fig. 2) and in Category B applications (Fig. 3), may simply further encourage some applicants to pursue the strategy of submitting many proposals. Intake limits, if imposed by funding agencies for individual applicants, or quota systems, could serve as a counter measure, thereby helping to contain system cost and reduce burden to reviewers.

In the context of small competitions, such as the comparison of review systems for 32 applications [14], the suggestion that all reviewers review all applications may often be feasible. This approach would not be operationally realistic in competitions with either large committees or large competitions where there is no meeting of a committee-as-a-whole (as in this Fellowship competition). For such large competitions, especially with low overall success rates, use of 5 reviewers per application is recommended (which would also facilitate tie-breaking during discussion).

A true estimate of the probability distribution of the reviews for each application could be obtained in the theoretical case of a very large (or infinite) number of reviewers. However, sampling this *a priori* unknown distribution by a finite number of reviews per application (e.g., N from 1 to 21, as in the current study, or all actual competitions), will lead to false results some of the time. While the quality of the estimation would improve with increased reviewers, ad infinitum (e.g., as in Fig. 4, where kappas progressively increase with N_reviewers_), practical and resource constraints impose severe limits to reviewer numbers.

These findings and conclusions, based on a single set of applications from a single program, may have limited applicability to other funding or selection scenarios. It would be especially useful to have validation from research grant competitions involving multiple applicants per proposal (e.g., research grants for large teams) and from competitions involving non-uniform financial requests, where considerations of potential return on investment may be important considerations for reviewers. Nevertheless, this study does provide a basis for recommending that research funding agencies and journals avoid extremes in numbers of reviewers per application. Having too few reviewers (i.e., N ≤ 3) results in excessive decision inconsistency. On the other hand, while theoretically preferable, having larger numbers of reviewers (N > 5) yields only modest improvement in consistency but does entail substantial increase to cost, burden and difficulty in recruiting additional reviewers. Five reviewers per application represents a practical trade—off, in terms of balancing increased decision consistency against incremental cost, as well as minimizing large random effects in decision outcomes and improving efficiency of the decision making process.

## Supporting Information

S1 Data Reviewer scores for 100 Fellowship applications (9 independent scores per application). Anonymised data from Canadian Institutes of Health Research spring 2013 post-doctoral Fellowships competition for biomedical applicants.
(CSV)

S1 File R code used for analyses and graphics. Some annotations and cropping in final images (e.g., Fig. 2 category boundaries and category labels, Fig. 3 arrows) done with graphic image editing software.
(R) Click here for additional data file.
